# Digitally managed larviciding as a cost-effective intervention for urban malaria: operational lessons from a pilot in São Tomé and Príncipe guided by the Zzapp system

**DOI:** 10.1186/s12936-023-04543-0

**Published:** 2023-04-06

**Authors:** Arbel Vigodny, Michael Ben Aharon, Alexandra Wharton-Smith, Yonatan Fialkoff, Arnon Houri-Yafin, Fernando Bragança, Flavio Soares Da Graça, Dan Gluck, João Alcântara Viegas D’Abreu, Herodes Rompão

**Affiliations:** 1 ZzappMalaria, Menachem Begin 23, Tel Aviv, Israel; 2 School of Hygiene and Tropical Medicine, ZzappMalaria, London, Thailand; 3 ZzappMalaria, São Tomé, São Tomé and Príncipe; 4 Independent researcher, Tel Aviv, Israel; 5 Ministry of Health, São Tomé, São Tomé and Príncipe

**Keywords:** Urban malaria, Vector control, Larval source management, Digitally managed larviciding

## Abstract

**Background:**

Once a mainstay of malaria elimination operations, larval source management (LSM)—namely, the treatment of mosquito breeding habitats–has been marginalized in Africa in favour of long-lasting insecticidal nets (LLINs) and indoor residual spraying (IRS). However, the development of new technologies, and mosquitoes' growing resistance to insecticides used in LLINs and IRS raise renewed interest in LSM.

**Methods:**

A digitally managed larviciding (DML) operation in three of the seven districts of São Tomé and Príncipe (STP) was launched by the Ministry of Health (MOH) and ZzappMalaria LTD. The operation was guided by the Zzapp system, consisting of a designated GPS-based mobile application and an online dashboard, which facilitates the detection, sampling and treatment of mosquito breeding sites. During the operation, quality assurance (QA) procedures and field management methods were developed and implemented.

**Results:**

12,788 water bodies were located and treated a total of 128,864 times. The reduction impact on mosquito population and on malaria incidence was 74.90% and 52.5%, respectively. The overall cost per person protected (PPP) was US$ 0.86. The cost varied between areas: US$ 0.44 PPP in the urban area, and US$ 1.41 PPP in the rural area. The main cost drivers were labour, transportation and larvicide material.

**Conclusion:**

DML can yield highly cost-effective results, especially in urban areas. Digital tools facilitate standardization of operations, implementation of QA procedures and monitoring of fieldworkers’ performance. Digitally generated spatial data also have the potential to assist integrated vector management (IVM) operations. A randomized controlled trial (RCT) with a larger sample is needed to further substantiate findings.

**Supplementary Information:**

The online version contains supplementary material available at 10.1186/s12936-023-04543-0.

## Background

Targeting water bodies in which mosquitoes breed was the mainstay of many malaria control operations in the 1930s and 1940s, often resulting in complete elimination of local malaria transmission. A notable example is an operation in eastern Brazil led by the epidemiologist Fred Soper, where the invasive *Anopheles gambiae* was eliminated from the country within less than two years. Soper, who was known for his thoroughness, emphasized close monitoring and clear assignment of responsibility to individuals [1]. Indeed, the success of LSM operations depends greatly on the proportion of water bodies that can be identified and treated (intervention coverage) [2]. Unfortunately, attempts to introduce LSM, and specifically larviciding, to sub-Saharan Africa were often met with operational difficulties that led to limited coverage and an insufficient reducing of mosquito populations [3]. The current World Health Organization (WHO) guidelines recommend LSM as a supplementary intervention alongside LLINs and IRS, and only in areas where water bodies are “few, fixed, and findable” [4].

Nevertheless, several factors contribute to a renewed interest in LSM. First, it helps mitigate two of the main challenges faced by LLINs and IRS—outdoor biting behaviour and insecticide resistance [5]. This, because it affects mosquitoes at their aquatic stages through biological agents or physical mechanisms to which they are not expected to develop resistance [6]. In addition, LSM is potentially highly cost-effective in urban settings [7], making it an attractive solution in light of the growing rate of urbanization in sub-Saharan Africa [8] and of the spread in Africa of the invasive species *Anopheles stephensi* that thrives in cities [9]. Finally, new technologies, e.g., drones and artificial intelligence, can facilitate detection and treatment of water bodies [10]. Similarly, digital tools can promote data-based and data-driven interventions and improve the operational and managerial aspects of large-scale LSM operations.

One such tool is the Zzapp system, which was developed to address the operational challenges involved in large-scale malaria vector control operations. The system comprises (1) an online dashboard where areas for treatment are demarcated, and tasks are assigned and monitored by the district-level manager on a daily basis; and (2) a Mapbox-powered GPS-based mobile app that guides fieldworkers during implementation. The description below refers to the system’s capabilities in terms of larviciding management as used in the operation in STP. Other functionalities of the system, such as an artificial intelligence that plans IVM operations (e.g., targeting houses for IRS based on the location of the water bodies reported on the system) are not discussed here.

**Fig. 1:**
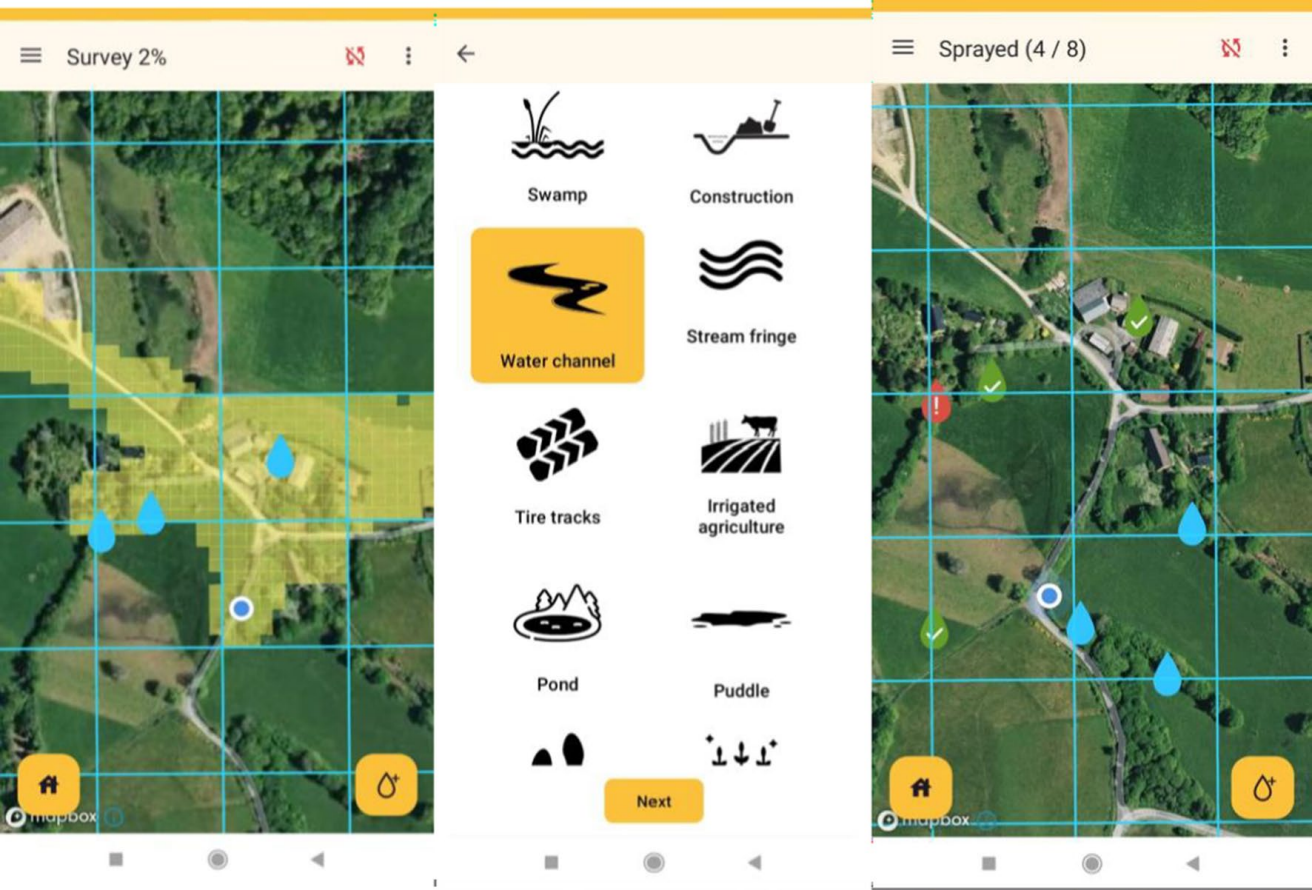
screenshots from the Zzapp mobile application. Left: Map view during mapping activity showing areas previously visited by the fieldworker highlighted in yellow. The blue circle indicates the current location of the fieldworker, and blue droplet icons indicate water bodies previously reported. Center: sample questions from the questionnaire completed by fieldworkers for every water body reported. Right: map view during treatment activity showing droplet icons corresponding to water bodies, color-coded by status (green: treated; red: indication of a problem preventing treatment; blue: untreated)

The system uses house location (based on satellite images) to define the areas that need to be treated within the districts that were chosen for the intervention. It then divides these areas into operational units. Managers working with the web-based dashboard assign these areas to fieldworkers, who are given specific tasks (scanning, treating, sampling or QA). Fieldworkers receive their tasks on mobile phones via a designated GPS-based application that can be used by any mobile phone with an Android operating system (version 7.0 or later) that has a GPS, a compass, a camera and more than 1 GB RAM (these requirements are met by most smartphones available in Africa today). The app supports the fieldworkers' activities based on the specific task they are implementing. In scanning tasks, it tracks the progress of the fieldworkers in the field, highlighting the areas that they have surveyed in order to ensure that the entire area has been scanned. In sampling and treatment tasks, the app guides the fieldworkers to the water bodies that are in need of treatment (Fig. 1). The app also enables fieldworkers to upload information about the water bodies they detect, sample and treat and to report the completion of their assignments.

**Fig. 2:**
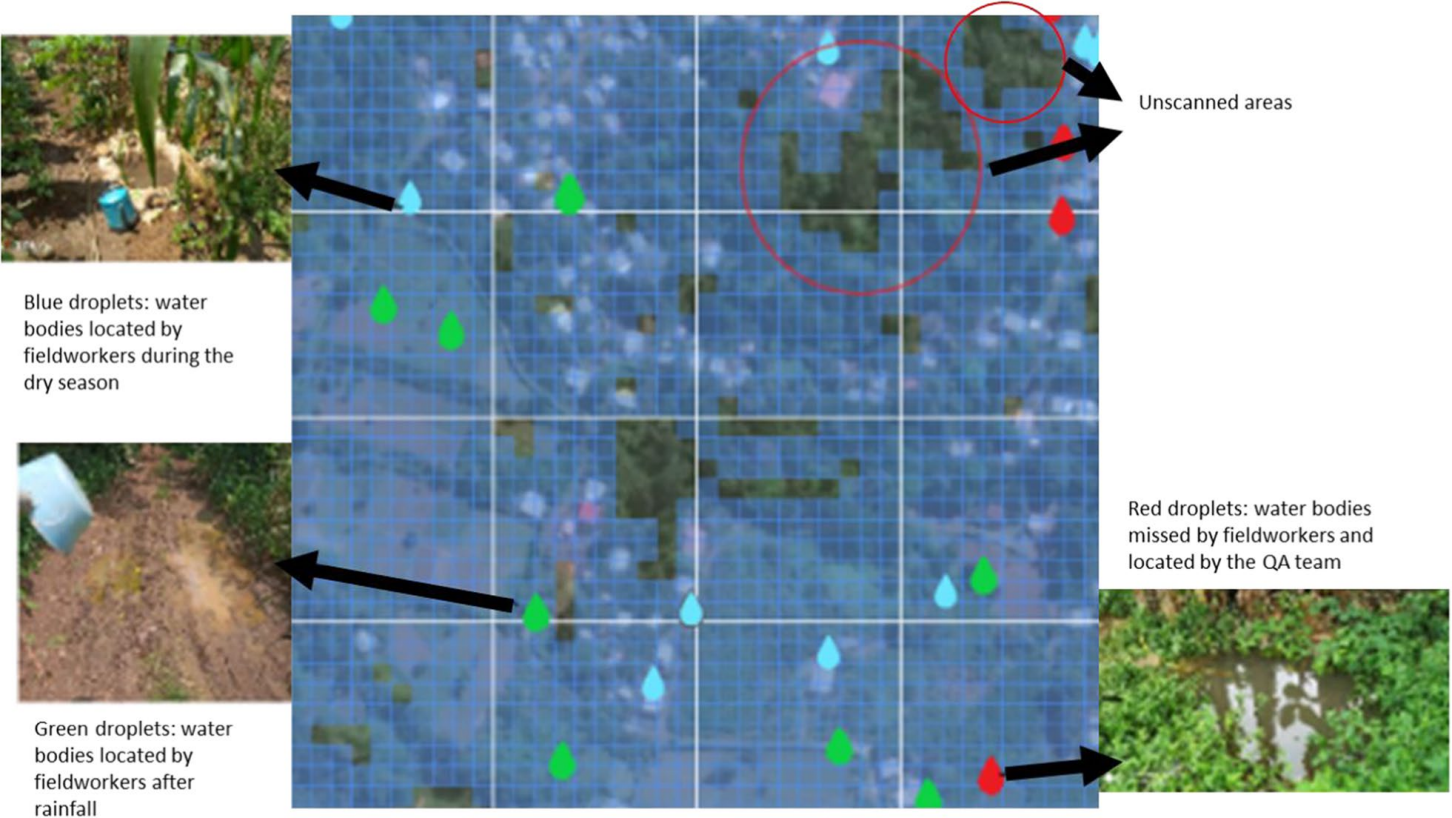
Coverage during the mapping phase. Blue squares indicate areas that were surveyed by fieldworkers (edited from a dashboard screenshot showing the village Blublu, Mé-Zóchi district, January 28, 2022). Maps data: ^©^2022 Google

All information is uploaded to the dashboard, allowing managers to monitor the operation. Based on the percentage of area that was scanned and the number of water bodies that were detected, sampled or treated, managers decide whether to approve the task’s completion, to ask the fieldworker to redo part or all of the task (e.g., scan an area that was missed), or to assign it to another fieldworker. The system also produces various reports on individual fieldworkers’ performance (e.g., the number of hours worked, the percentage of the area that was scanned and the number of water bodies that were found positive after being reported as treated) and on the overall coverage of villages (Fig. 2).

In a small-scale trial of the mobile app conducted in Obuasi, Ghana in 2018, fieldworkers guided by the app reported 28% more water bodies compared to a control team that mapped the same area using traditional methods [11]. The current article reports the results of an operation conducted in STP by ZzappMalaria LTD and the STP MOH.

## Methods

### Study site

**Fig. 3:**
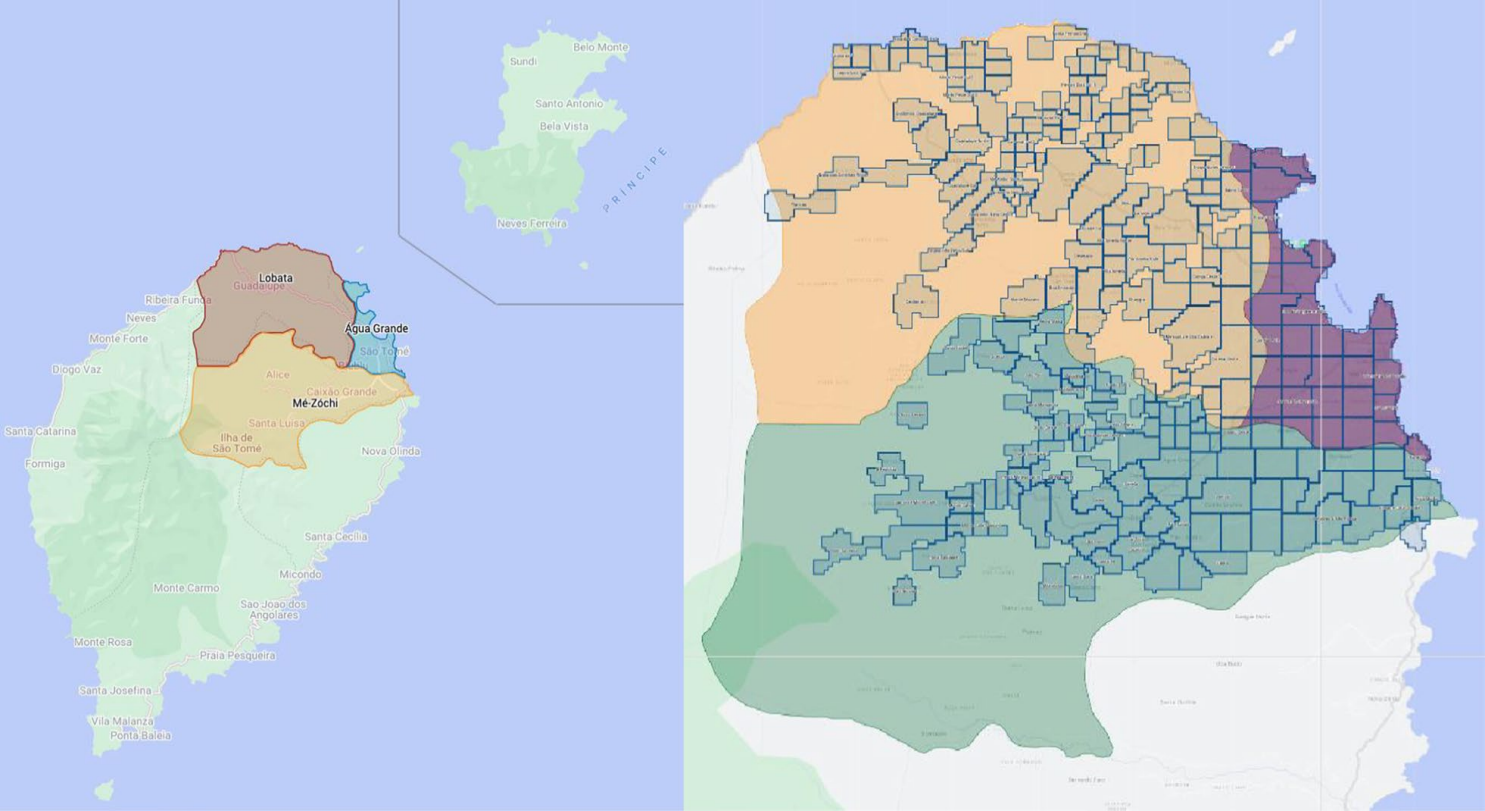
São Tomé and Príncipe. Left: Map of São Tomé and Príncipe, with the intervention districts Água Grande, Lobata and Mé-Zóchi highlighted. Right: Within the three districts, the Zzapp system marked the populated areas and divided them into 255 operational units, with a total area of 125.41 km^2^

The Democratic Republic of STP is an island country in the Gulf of Guinea that consists of two main islands, São Tomé and Príncipe. As of 2021, STP has an estimated population of 228,000 [12], more than 95% of whom live on the island of São Tomé. This 854-km^2^ island contains various climatic regions and has a prolonged rainy season that begins in September and lasts through May. The reported number of malaria cases in STP in 2020 was 1933, with an incidence of 8.7 per 1000.^9^ The larviciding pilot was performed in three districts: Água Grande (an urban district), Mé-Zóchi, and Lobata (rural and semi-urban), with a combined area of 243.5km^2^ (28% of the island of São Tomé’s area), and an estimated population of 166,500 people (73% of the country’s population). The districts of Cantagalo, Lembá, Caué and the autonomous island of Príncipe (rural and semi-urban), with a total estimated population of 61,500 people, were not included in the intervention and were therefore used as a control (Fig. 3). Note that the pilot is not well balanced, since the control area does not have an urban district, such as Água Grande. Hence, for robustness, the main results of the study are reported both with and without Água Grande.

While not part of the reported digitized larviciding intervention, ongoing vector control activities—including IRS, LLINs, drug distribution and community-based larviciding—continued to take place in all the districts.

The larviciding operation was divided into two phases: a mapping phase during which fieldworkers searched for water bodies, and a treatment phase in which water bodies were treated with larvicide on a weekly basis. The larvicide materials used were VectoBac^®^ G (granules) applied by hand and VectoBac^®^ WDG (water dispersible granules) applied as an aqueous solution. Both products contain the bacteria *Bacillus thuringiensis* var. israelensis (*Bti*), which produces toxins targeting a specific protein in the digestive tract of mosquito and black fly larvae, without any harmful effects on other insects or vertebrates.

Prior to the implementation, fieldworkers underwent a three-day training course, which included an overview of the malaria transmission cycle and the life cycle of the *Anopheles* mosquito; the objectives of the operation; guidance on the use of the mobile app in the execution of mapping and application of larviciding of water bodies (including large water bodies); personal safety; field practice; and a practical test. A designated team was trained by an entomologist from the MOH to sample water bodies for mosquito larvae and pupae. Sampling was performed at the beginning of the operation (i.e., prior to treatment of any water bodies) to determine the baseline positivity rate, and continued biweekly throughout the operation in the same villages sampled at the baseline. This team repeatedly sampled the same villages in which 150 water bodies testing positive for *Anopheles* larvae were identified prior to larvicide application (50 positive water bodies per district) in order to monitor the change in positivity over time.

**Fig. 4:**
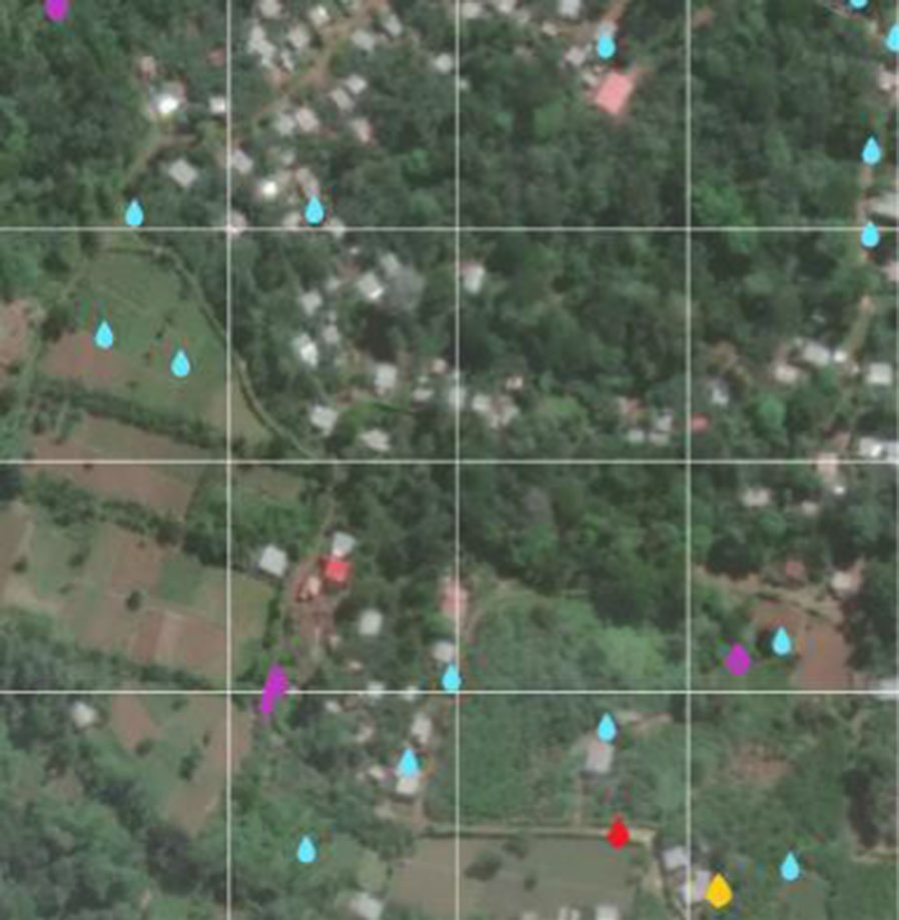
Coverage during the treatment phase. Blue droplets: treated water bodies. Purple droplets: water bodies located during the mapping phase but reported during the treatment phase as nonexistent (e.g., dried out). Orange droplets: water bodies skipped by the fieldworker. Red droplets: water bodies reported as treated but then found positive by the QA team (edited from a dashboard screen presenting the village Blublu, Mé-Zóchi district, January 28th, 2022)

Another group of fieldworkers, who were also trained to sample water bodies, served as a QA team. Their goal was to compliment the Zzapp system in ensuring that the entire area was scanned; that within this area all the water bodies were located; that all water bodies were treated properly (i.e., with the right amount of larvicide at the right frequency); and that all water bodies that appeared in the aftermath of rain were detected. QA was reached by rescanning certain areas (either by the QA team or by regular fieldworkers) and by sampling, for each fieldworker, a few treated water bodies in order to verify the proper application of larvicide (Fig. 4).

QA was done either randomly or based on underperformance detected with the aid of the dashboard. During the scanning phase, some of the workers achieved less than 50% coverage of the areas they were assigned to search. QA teams were sent to those areas to determine whether or not the low coverage was justified (e.g., because of inaccessibility). Based on the QA results, some fieldworkers were retrained. QA was also used during the treatment phase, where some water bodies were sampled after they were reported as treated. Dashboard reports revealed that 64% of the water bodies that were discovered to be positive 1–6 days after treatment (i.e., were either not treated properly or were falsely/mistakenly reported as treated) were attributable to 15% of the fieldworkers. These fieldworkers were retrained or reassigned to other tasks.

Toward the end of the operation, after realizing that the preset milestones for progress were not being met, the system was utilized to produce weekly reports to evaluate fieldworkers' progress with regard to working hours, number of areas assigned for scanning, level of scanning coverage within the assigned areas and the number of water bodies that were either missed or insufficiently treated. In addition, focus group discussions, in-depth interviews, field visits and informal discussions were carried out to enable better understanding of fieldworkers’ expectations and implementation challenges. As a result, the employment structure was rearranged, new agreements specifying working hours and tasks were signed, a bonus system that awarded cash handouts to outstanding workers was established, and workers were provided with daily lunches. These changes correlated to an increase in productivity of 26%.

The operation was piloted to test the Zzapp system in preparation for a nationwide operation in STP and was not designed as a cluster-RCT. Its effects were measured according to two entomological and one epidemiological criteria: (1) effect on larvae/ pupae and adult mosquitoes; and (2) effect on malaria incidence. Effect on larvae and pupae was measured through sampling of water bodies performed by fieldworkers trained by an entomologist from the MOH. In each sampling event, five scoops of water were taken from the water body and the larvae and pupae were counted for each scoop, based on their stage of development: *Anopheles* 1st-2nd instar larvae, *Anopheles* 3rd-4th instar larvae, *Culex/Aedes* 1st-2nd instar larvae, *Culex/Aedes* 3rd-4th instar larvae, and pupae (all species). Sampling was performed at the beginning of the operation (i.e., prior to treatment of any water bodies) to determine the baseline positivity rate, and continued biweekly throughout the operation in the same villages sampled at the baseline. Additionally, the QA team sampled a few water bodies treated by each fieldworker in order to verify proper treatment.

### Impact on adult mosquitoes

The intervention's effect on the adult mosquito population relies on the MOH's routine entomological sampling conducted biweekly by the STP Ministry of Health, in two locations in each of the country seven districts. The collections include both CDC light traps and human landing collections (HLC), indoors and outdoors. Based on historical data, indoor HLC and indoor and outdoor light traps capture a low number of mosquitoes. For this reason, only outdoor HLC data were used for the analysis.

For each collection point, the ratio between the after and before was used as an estimator of mosquito population increase in that community. The median of the ratios in all the intervention communities was used as a robust estimator of the increase in the entire intervention area. The increase in the control was estimated in the same way. The ratio between the increase in the intervention area and the increase in the control area is the estimator of the intervention's impact. Mean ratios, confidence intervals and T-test results were also calculated. Since the intervention and control area are not well balanced (because the intervention area contains the urban Água Grande district), all calculations were repeated without Água Grande. Finally, because of the small number of sampling locations and the difficulty of assuming normal distribution of the ratio, the nonparametric Mann–Whitney p-value was also calculated.

### Impact on malaria incidence

The intervention's impact on malaria incidence relies on official malaria case data, which in STP is routinely collected by the MOH, based on weekly reports from health facilities that attribute each malaria case to a location at the village level. The malaria incidence per 10,000 people during in the entire intervention area and the entire control area were calculated, both in the before-intervention period (weeks 1–49 of 2021) and in the after-intervention period (weeks 1–19 of 2022). The ratio between the intervention and control after-before ratio is the estimator of the intervention’s impact. Confidence intervals were calculated based on village-level incidence data. Because of the difficulty to calculate standard deviation for ratios, especially because many of the villages had zero cases, bootstrapping confidence intervals were calculated, using the Monte Carlo algorithm of case resampling. Note that mosquitoes travel from village to village, and therefore the villages are not entirely independent from one another.

For the intervention area, an additional calculation was done in which the urban district of Água Grande was excluded, in order to make the intervention and control better balanced. To reflect the per-district effect, which suits better to vector control intervention, as the districts are more likely to be independently distributed compared to villages, a Man-Whitney p value was used, excluding districts with less than 10 cases in the entire “before” period. The caveat of this approach is the very small sample size (7 districts rather than of 533 villages).

## Results

### Operational findings

**Fig. 5:**
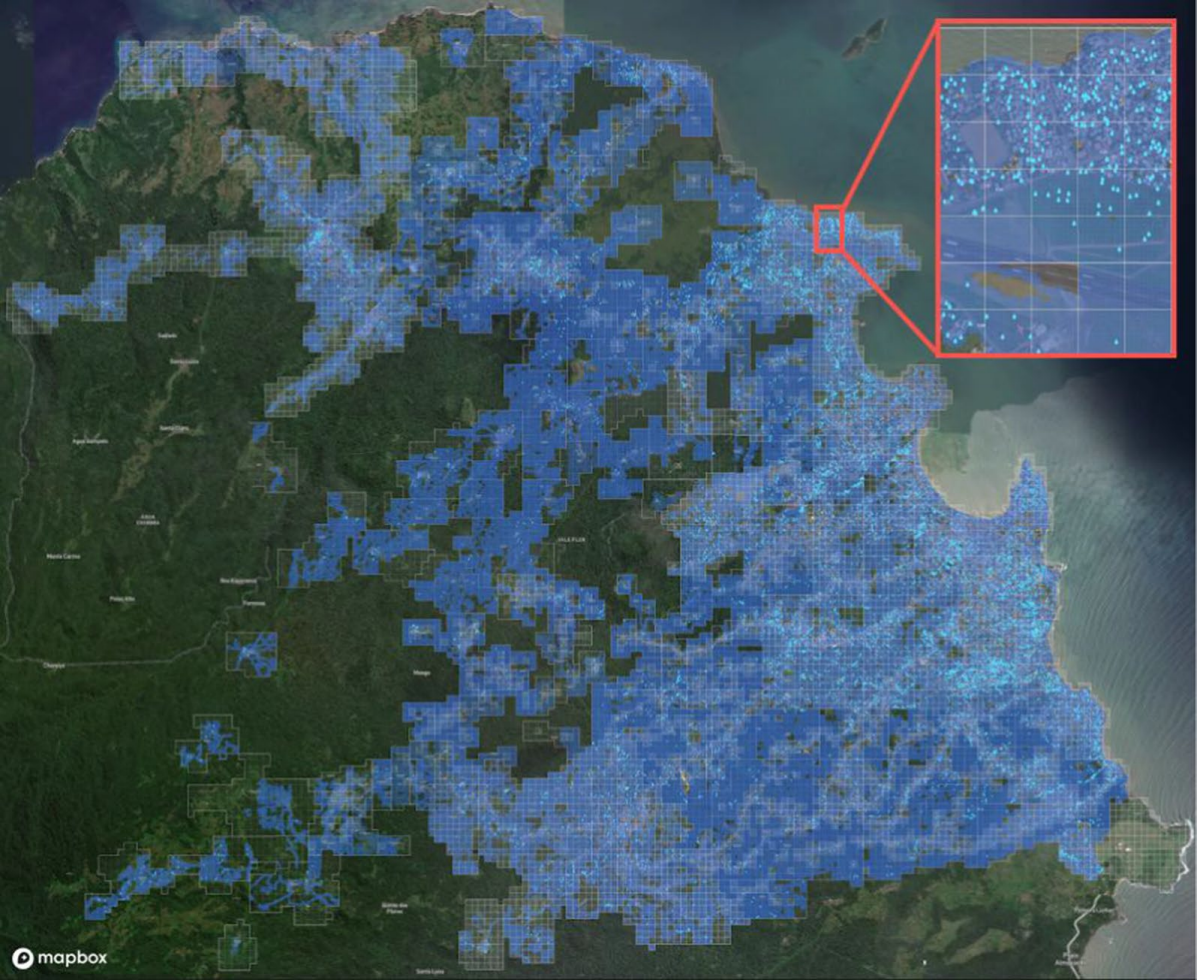
Coverage obtained in mapping activities. The white polygons indicate the area identified for the larviciding intervention. Areas visited by fieldworkers during the mapping stage are highlighted in blue (at a resolution of 10 m^2^). Blue droplet icons mark water bodies reported by fieldworkers

The total area visited by fieldworkers during the ground survey was 90.8 km^2^ (Fig. 5). A total of 12,788 water bodies were reported on the system. These water bodies were treated a total of 128,864 times and sampled 31,353 times. A total of 28,250 “issues” regarding them (e.g., disappearance or lack of accessibility) was reported on the system.

#### Impact on larvae and pupae

**Fig. 6:**
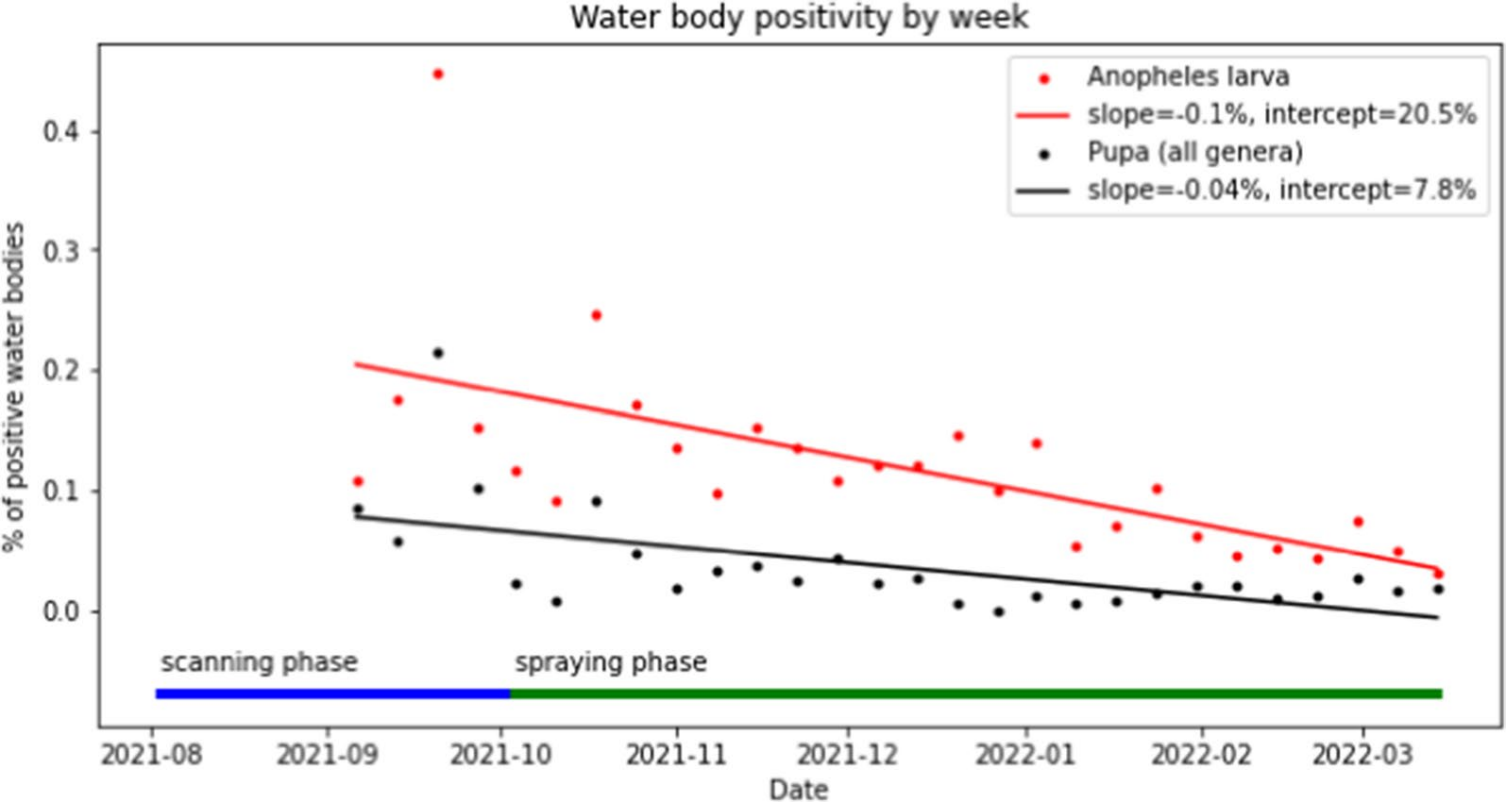
Larva and pupa positivity over the course of the operation

Overall, 31,353 water body samples were conducted throughout the operation, showing a decrease of 61.64% in the *Anopheles* larva positivity rate during the treatment phase, from 19.42% before the first treatment to 7.44% after 1/12/2022, and a reduction of 81.84% in the pupa positivity rate, from 9.24% before the first treatment to 1.67% after 1/12/2022 (Fig. 6). Notwithstanding this trend, some water bodies remained positive even after the treatment phase, either because they were treated improperly, skipped during the treatment phase, or appeared after the mapping phase.

Table 1 presents the positivity of water bodies before and during the intervention. The Zzapp system helps to analyse the positivity of water bodies by type and characteristics before the intervention, and to identify water bodies that remain positive after treatment (Table 1, Additional file 2: Appendix S2). This knowledge can be used in cross-sector collaborations, for example, to notify the municipality that channels are one of the main sources of mosquitoes. It can also serve operational purposes, such as understanding whether fieldworkers are properly treating polluted water bodies (Additional file 2: Appendix S2).

**Table 1:** Distribution of water bodies according to type

Water body type	Number of water bodies	Baseline (prior to treatment)				Treated water bodies			
		Number of samples	Pupa positive samples	Positivity rate (%)	95% CI Wilson (%)	Number of samples	Pupa positive samples	Positivity rate	95% CI Wilson (%)
Puddle	4412 (35%)	215	14	6.51	5.11–8.26%	4695	67	1.43	1.27–1.60%
Channel	2920 (23%)	324	26	8.02	6.73–9.54%	3834	41	1.07	0.93–1.24%
Swamp	2041 (16%)	121	6	4.96	3.42–7.14%	2687	32	1.19	1.01–1.40%
Construction	906 (7%)	75	21	28.00	23.45–33.05%	1250	22	1.76	1.45% -2.14%
Pond	626 (5%)	49	4	8.16	5.21– 12.57%	934	2	0.21	0.11–0.41%
Tracks	574 (4%)	24	2	8.33	4.43–15.14%	677	7	1.03	0.73–1.46%
Agriculture	411 (3%)	11	3	27.27	16.79–41.07%	299	6	2.01	1.38–2.91%
Fringe	389 (3%)	38	4	10.53	6.74–16.07%	484	3	0.62	0.36–1.05%
Others	509 (4%)	8	0	0.00	0.00–9.76%	588	8	1.36	0.98–1.88%
Total	12,788 (100%)	865	80	9.25	8.37–10.21%	15,448	188	1.22	1.14–1.30%

**Fig. 7:**
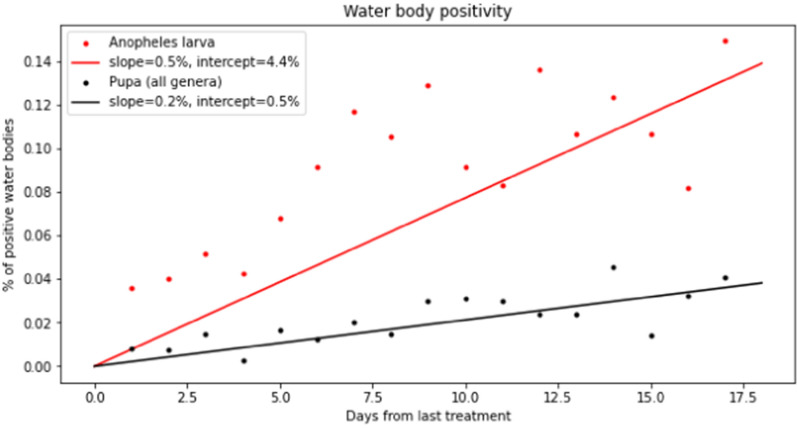
Sampling of water bodies by the QA team. Note that the baseline positivity (before treatment) for larvae is 19.3% and for pupae is 9.2%. Even after 17 days, the treatment has some impact on water body positivity for *Anopheles* mosquitoes

An additional factor accounting for positivity of water bodies may have been insufficient frequency of treatment. Figure 7 shows the correlation between positivity of water bodies and the number of days since the last treatment. In this pilot, the minimum time between treatment events was set as five days, the target interval as seven days and the maximum interval as 14 days, after which the system would alert the operation administrator through the dashboard. In the operation, the average interval between visits was 10**.**8 days, which may explain the positivity of some water bodies.

### Impact on adult mosquitoes

Indoor collections produced low *Anopheles* counts compared with outdoor collections. The monthly averages of *Anopheles* mosquitoes collected in 2021, for all collection points combined, was 0.8 (indoors) vs. 18 (outdoors) for HLC (Table 2). Only the outdoor collections were used, to reduce statistical noise.

**Table 2:** Total Anopheles outdoor HLC, before and after intervention

District	Community	Outdoor *Anopheles* HLC, before intervention, monthly average	Outdoor *Anopheles* HLC, after intervention, monthly average	Ratio after/before
Intervention communities				
Água Grande	Praia Gamboa	42.78	11.67	0.27
Água Grande	Madre De Deus	2.78	0.33	0.12
Mé-Zóchi	Cidade De Cruzeiro	1.00	0.00	0.00
Mé-Zóchi	Cidade De Praia Melão	14.22	1.00	0.07
Lobata	Micolo	23.22	28.33	1.22
Lobata	Conde	19.78	16.00	0.81
	Median			0.2
Control communities				
Lembá	Cidade Das Neves	15.33	32.67	2.13
Lembá	Ponta Figo	5.22	0.33	0.06
Cantagalo	Ribeira Afonso	45.78	12.00	0.26
Cantagalo	Zandrigo	9.33	50.67	5.43
Príncipe	Porto Real	22.33	21.67	0.97
Príncipe	Rua Dos Trabalhadores	23.11	18.00	0.78
Caué	Angolares	0.33	0.00	0.00
Caué	Emolve	33.89	26.33	0.78
	Median			0.78

The average after-before monthly ratio was 0.41 (95% CI 0.01–0.81, median = 0.2) and 1.3 (95% CI 0–2.54, median = 0.78) for intervention (n = 6) and the control (n = 8) areas, respectively (p = 0.11). The median relative change (intervention vs. control) was − 74.9% (95% CI − 100%, − 30%, Mann–Whitney p = 0.26). For the intervention area without the urban district of Água Grande (n = 5), the mean was 0.51 (95% CI 0–0.52, p = 0.15).

The level of significance of the results is consistent with what is considered acceptable for clinical pilot studies [13, 14], but requires further confirmation from a well-powered and balanced clustered cluster-RCT. It is important to note, however, that this result remains robust in other analysis methods—including indoor collections data and using light traps instead of HLC.

#### Impact on malaria incidence

The after-before ratio in the control areas was 3.57 and in the intervention area 1.7 (ratio = 0.475, p = 0.006), which is a 52**.**5% reduction. The results remain similar when excluding the urban area of Água Grande from the sample (ratio = 0.47 p = 0.008). P-value of the district-based Mann–Whitney test is 0.35 (n_intervention = 3, n_control = 3). Note that the district of Príncipe was excluded from the Mann–Whitney test, because it had fewer than 10 malaria cases in the “before” period. All the other districts had more than 40 cases each in this period (Table 3).

**Table 3:** Before and after, control and intervention ratios

	Before mean (95% CI)	After mean (95% CI)	Ratio
Intervention: average incidence per 10,000 per week	2.83 (2.34–3.33)	4.82 (4.03–5.63)	1.70 (1.39–2.07)
Control: average incidence per 10,000 per week	0.86 (0.66–1.06)	3.08 (2.32–3.85)	3.57 (2.14–5.90)
Intervention, without Água Grande: weekly average incidence per 10,000	4.42 (3.51–5.32)	8.12 (6.59–9.66)	1.84 (1.28–2.21)

### Cost

The total cost of the operation, including a two-month mapping stage and a 5**.**5-month larviciding stage, was US$ 143,821. Cost categories are detailed below. The main cost drivers were labour, transportation and larvicide material (similar to Worrall et al. [15]). The overall cost of the operation per person protected (PPP) was US$ 0.86. Cost varied significantly with population density (Table 4).

**Table 4:** Cost categories divided between urban and rural areas (US$)

	Urban and Rural		Urban		Rural	
	Cost	%	Cost	%	Cost	%
Labour—fieldwork	52,694	36.64	14,333	34.87	38,361	37.35
Transportation (taxis)	32,025	22.27	7545	18.35	24,480	23.83
Larvicide material (*Bti*)	15,540	10.81	7380	17.95	8160	7.94
Labour—management	10,884	7.57	2960	7.20	7923	7.71
Meals	5112	3.55	1390	3.38	3721	3.62
Personal protective equipment (e.g., boots, reflective vests)	4974	3.46	1353	3.29	3621	3.53
Mobile devices	3119	2.17	848	2.06	2270	2.21
Internet	1800	1.25	490	1.19	1310	1.28
Information, education and communication activities (TV and radio ads, posters, opening ceremony)	3327	2.31	905	2.20	2422	2.36
Office supplies and tools	399	0.28	109	0.27	291	0.28
Zzapp: campaign setup, cloud services and software adjustment	4248	2.95	1156	2.81	3092	3.01
Zzapp: training for management/field staff	6530	4.54	1777	4.32	4753	4.63
Zzapp: weekly reports and operational insights	3169	2.20	863	2.10	2306	2.25
Total	143,821	100.00	41,109	100.00	102,710	100.00

**Fig. 8:**
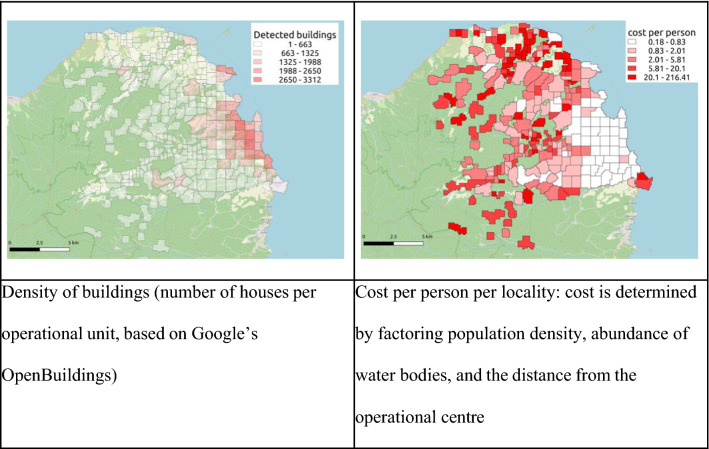
Comparison of population density (based on number of buildings per km^2^; left) and cost PPP per locality (right)

Of the total scanned area, 12**.**87% (16**.**15 km^2^) was urban (> 1,500 structures per square km, based on Open Buildings dataset) [16], in which an estimated 56**.**31% of the total intervention population (93,762 people) live. For higher resolution of the correlation between population density and cost PPP, see Fig. 7. According to data from the mobile application, 27**.**2% of workdays and 47**.**5% of treatment events took place in these urban localities. The cost in urban areas was an estimated US$ 41,109, and US$ 0.44 PPP, and the cost in rural areas was an estimated US$ 102,710, and US$ 1.41 PPP (see Fig. 8). Additional file 1: Appendix S1 presents a more detailed calculation of cost and the cost saving that could be achieved by using operation-owned cars instead of taxis.

## Discussion

The goal of this study was to demonstrate the potential of digital tools to facilitate the strategization, implementation and monitoring of wide-scale LSM operations. Findings from the operation reported here indicate the high cost-effectiveness of DML. The effectiveness that was achieved—52.5% reduction of malaria incidence—is comparable to that reported in studies measuring the effectiveness of LLINs (45% [17]) and IRS (18% [18]). The cost was US$ 0**.**86 per person protected per 6 months compared with US$ 0.695 [19] and US$ 6.19 [20] for LLINs and IRS, respectively. In urban and semi-urban areas, the cost of DML was significantly lower than that of LLINs: $0.44 per person protected per 6 months, compared with US$ 0.695. Note that the results indicate that the intervention is also effective in rural areas.

The advantage of DML may be even greater when considering the room for improvement (see elaboration in Additional file 1: Appendix S1). While the distinction between urban and rural areas in the context of malaria is not always clear [21], AI tools that count houses, such as the Zzapp system or Google’s Open Buildings project, enable estimation of operational costs for different locations (Fig. 8), which can help policymakers allocate malaria budgets between DML and other interventions in a cost-effective way. Crucially, digitization enables effective monitoring of the operation, making operations more standardized and replicable.

Note that some of the results, especially at the district level, were not statistically significant, perhaps because of the low number of districts. Moreover, the districts were not well balanced between urban and rural areas. There is therefore a need to confirm the findings via a well-designed cluster RCT, which will also examine the incremental value of DLM compared with conventional larviciding operations.

The cost-effectiveness of DML has the potential of being enhanced via the use of new tools. For example, detection of water bodies through drone or satellite imagery using AI can be used to optimize the detection and treatment of water bodies [22, 23], and analysis of weather conditions and patterns may help to choose the best timing for interventions.

It is important to note that during DML operations, a large amount of data regarding the location and other parameters (e.g., type or positivity level) of mosquito breeding sites is collected. This information can be used not only for the immediate functional proposes of LSM operations, but also to add to the knowledge about vector dynamics more generally. With regard to both, DML can play a crucial role in mitigating the risk posed to African countries by the invading species *Anopheles stephensi*. This vector, unlike the native African species, is capable of breeding in manmade water containers that are abundant in cities, in this way posing a threat to large populations that had previously been less affected by malaria [9]. A*nopheles stephensi* has also shown resistance to the insecticides recommended by the WHO in LLINs and IRS, and its control therefore focuses on strategies that do not include insecticides, such as LSM [24]. Digitization not only can facilitate such vector control operations, but can also support national and regional surveillance efforts.

Moreover, spatial modeling and data about the location of water bodies can optimize the use of other methods, e.g., by recommending which houses should be treated with IRS or where to place attractive targeted sugar baits (ATSBs) [25]. Thus, digitization may enable the long-sought, but seldom implemented, IVM operations, which have been recommended in the context of *An. Stephensi* [26] and vector control more broadly [27].

The key for all the above is efficient monitoring mechanisms that provide reliable and granular data in real time. During operations, monitoring enables progress-tracking, evaluation of workers’ performance, flagging of areas requiring extra attention, and expansion of successful interventions. Monitoring also facilitates tracking of expenses, thereby both helping to reduce operational costs and providing clear, detailed and precise accountability reports to stakeholders. Finally, close monitoring strengthens the robustness of results, yielding reliable data-based insights and recommendations for future research and interventions. Digitization facilitates the pooling and analysis of various malaria data and on multiple levels—from the location of a water body in a village to the average distance to hospitals in a given district, from transportation costs to community acceptance. Aggregating this information into a single, spatial-based platform can significantly improve vector control operations, to the extent of reproducing the results of historical LSM operations: nationwide elimination.

## Conclusion

LSM is one of the oldest methods for fighting malaria; with the use of modern tools it may also be one of the most cost-effective. Larviciding is safe, simple and proven, and adds an intervention that is synergistic with adulticiding methods such as IRS and LLINs. While currently considered by many as a secondary vector control method, LSM holds the potential of becoming the cornerstone intervention of large-scale and cost-effective vector control operations. Properly planned and thoroughly executed LSM operations target the problem from the root, and provide valuable information about the location, type and positivity of water bodies, which can serve as a basis for optimizing other vector control interventions. Digitization facilitates all aspects of LSM operations: from planning, through execution to monitoring. It eases the work of managers and fieldworkers and presents a clear and reliable picture of operations' progress, expenditure and outcomes, which can easily be shared with stakeholders and the community. Further research and experimentation is needed to fully exhaust the possibilities of DML and its transformation into the basis for a full-fledged, digital IVM (dIVM) system.

## Supplementary Information

**Additional file 1: Table S1.** Rural vs. urban localities in the intervention area. **Table S2. **Estimated transportation costs (US$).

**Additional file 2:** **Table S3.** Distribution of water bodies according to type. **Table S4. **Logistic regression of characteristics of water bodies and positivity before treatment. **Table S5. **Logistic regression of water bodies characteristics and positivity after treatment. **Fig**.** S1. **Water body positivity based on type.

## Data Availability

The datasets generated during and/or analysed during the current study are available from the corresponding author upon reasonable request.
