# Supplementary material for: Digitally managed larviciding as a cost-effective intervention for urban malaria: operational lessons from a pilot in São Tomé and Príncipe guided by the Zzapp system
Source: Malar J. 2023 Apr 6;22:114. doi: 10.1186/s12936-023-04543-0 (PMC10080920; doi:10.1186/s12936-023-04543-0)
Supplement: Supplementary file 1 — Additional file 1: Table S1. Rural vs. urban localities in the intervention area. Table S2. Estimated transportation costs (US$). [file 12936_2023_4543_MOESM1_ESM.docx]

Appendix 1. Elaboration of cost

Following Worrall’s ingredients approach^[[1]](#footnote-1)^ the overall financial cost of the operation was calculated by identifying each activity involved in the operation from the perspective of the intervention provider, and summing its costs. Data sources used for the cost estimation include expense reports maintained by the STP MOH, National Center for Endemic Diseases (Centro Nacional de Endemias; CNE) and ZzappMalaria, fieldworker attendance logs, transportation usage logs, and mobile device inventory logs. Access to the mobile application, training for project managers, and ongoing support for the Zzapp system were provided by ZzappMalaria at no cost, and were calculated based on the cost of labour, flights, and software cloud that were directly related to this operation. To each cost component, Zzapp added 60% to reflect profit and indirect cost. Office space was available at the CNE prior to commencement of the operation and was provided at no cost, and is thus excluded from the estimate. Research costs that would not normally be required for implementation of the larviciding intervention (e.g., increased entomological monitoring or field visits by the ZzappMalaria team) were also excluded. Expenses were converted to USD based on the exchange rate at the date of payment or using the average exchange rate in the relevant period.

Capital costs were calculated based on the predicted duration of use of budget items expected to last longer than the duration of the operation (7.5 months in total). The only capital cost was for the purchase of mobile devices for use by the fieldworkers. PPE and other equipment costs were not considered capital costs due to their high replacement rates.

Costs were further divided into those associated with operating in urban areas vs. rural areas (Table 4 in the main paper). Each locality was defined as urban or rural based on the number of structures per km^2^ (>1,500), which was calculated using Google’s Open Buildings dataset.^^[[2]](#endnote-1)^^ Data from the Zzapp mobile application were then used to determine the number of workdays spent in urban vs. rural localities, out of the total workdays registered. Labour costs and derivative costs, including management, equipment and supplies, internet usage, and information, education and communication (IEC) activities were divided into urban or rural based on the workdays ratio. Larvicide usage was divided based on data from the Zzapp application, which records individual treatment events by water body location and size. Surprisingly, water bodies in urban and rural area had similar size distribution; furthermore, the number of water bodies per km^2^ was higher in the urban areas. Transportation costs were divided based on daily transportation logs.

The financial cost PPP was calculated by dividing the total cost of the operation by the estimated population size in the three intervention districts, as obtained from official government statistics. The urban and rural population sizes were estimated by determining the total number of structures in each category, as described above, and multiplying the resulting ratio by the total intervention population (assuming similar occupancy per structure across the intervention districts).

**Spatial mapping of costs**

The cost of larviciding varies according to the prevalence of water bodies (which affects the amount of labour and Bti); population density within villages (which affects the amount of area to be scanned); and the distance between the villages and the operational center (which affects the consumption of fuel and fieldworkers’ transportation time). Table S1 compares various parameters of rural vs. urban localities in the intervention area in the operation in STP.

**Table S1. Rural vs. urban localities in the intervention area.**

|  | Urban | | Rural | |
| --- | --- | --- | --- | --- |
|  | # | Of total | # | Of total |
| Localities | 26 | 9.39% | 251 | 90.61% |
| Total area | 16.15 km^2^ | 11.61% | 122.92 km^2^ | 88.39% |
| Water bodies reported | 5,464 | 42.73% | 7,324 | 57.27% |
| Houses | 46,198 | 55.27% | 37,391 | 44.73% |
| Estimated population | 93,762 | 56.31% | 72,738 | 43.69% |
| Water body treatment events | 61,208 | 47.49% | 67,656 | 52.50% |
| Workdays | 1,341 | 27.17% | 3,594 | 72.83% |

Fig. 8 (in the main paper) presents a map of costing, based on data from the app’s log to factor the labour per village and the distance between villages to the operational center in order to assess the cost per location. As the map shows, the cost varies, with a few “villages” (actually lone houses and the isolated Voice of America radio station in the southwest) costing more than $200 PPP. It is important to note that while this tool is meant to assist managers in assessing costs and prescribing the appropriate interventions to each area, it is by no means intended to discriminate against any locality or any population. Areas for which larviciding is too expensive must be treated with alternative methods that do not require frequent visits—e.g., IRS and window screening—and are therefore less costly.

**Options for cost reduction**

A significant improvement of cost-effectiveness could be achieved by treating the water bodies during the scanning phase rather than in a separate treatment phase. Doing so is expected to prolong the impact of the operation with minimal additional cost (although it interferes with establishing an entomological baseline to monitor the operations results).

Due to the limited duration of the pilot, use was made of taxis instead of procuring cars, which significantly increased the cost of transportation. In preparation for expansion of the pilot to cover the entire island of São Tomé, we conducted a cost estimate for transportation using cars purchased for the project. Used vehicles suitable for the project requirements and the terrain on the island were searched online to determine availability and estimate cost. Logs from thse mobile app were analyzed to determine the typical distance traveled per day by the teams, the number of cars that would be needed to make the number of trips observed, and the number of kilometers traveled. Gas mileage was estimated based on averages published online for the cars selected. Maintenance and insurance costs were estimated by managers in São Tomé based on their experience with similar cars. Driver salaries were determined based on the rate paid by the MOH for drivers. Assuming a 10-year usable life for the cars, the estimated cost including fuel and salary for drivers was estimated to be between US$ 22,045 per year, and 14,772 for a period of 7.5 months (the duration of the trial)—less than half of what was actually spent (Table S2).

**Table S2** Estimated transportation costs (US$)

| **Capital Costs** | | | | | |
| --- | --- | --- | --- | --- | --- |
|  | Cost per unit | Units | Usable life (years) | Cost per year | Cost per 7**.**5 months |
| Vehicles | 23,000 | 3 | 10 | 6,900 | 5,175 |
| **Recurrent Costs** | | | | | |
|  | Cost per unit | Units | Period | Cost per year | Cost per 7.5 months |
| Fuel | 1.3 | 25/day | Day (20/month) | 7,800 | 4,875 |
| Drivers | 7.91 | 3/day | Day (20/month) | 5,695 | 3,559 |
| Maintenance | 350 | 3 | Year | 1,050 | 788 |
| Insurance | 200 | 3 | Year | 600 | 375 |
| Total | .. | .. | .. | 22,045 | 14,772 |

1. Worrall E, Fillinger U. Large-scale use of mosquito larval source management for malaria control in Africa: a cost analysis. Malar J. 2011;10:1-21 [↑](#footnote-ref-1)
2. https://sites.research.google/open-buildings/ [↑](#endnote-ref-1)
