# Supplementary material for: Digitally managed larviciding as a cost-effective intervention for urban malaria: operational lessons from a pilot in São Tomé and Príncipe guided by the Zzapp system
Source: Malar J. 2023 Apr 6;22:114. doi: 10.1186/s12936-023-04543-0 (PMC10080920; doi:10.1186/s12936-023-04543-0)
Supplement: Supplementary file 2 — Additional file 2: Table S3. Distribution of water bodies according to type. Table S4. Logistic regression of characteristics of water bodies and positivity before treatment. Table S5. Logistic regression of water bodies characteristics and positivity after treatment. Fig. S1. Water body positivity based on type. [file 12936_2023_4543_MOESM2_ESM.docx]

**Appendix 2: Correlation between water body characteristics and presence of aquatic stage mosquitoes**

When fieldworkers report the location of a water body for the first time, they also upload information about its type, size, pollution status and other characteristics. The positivity of water bodies at the baseline phase may be used to target larviciding, to plan additional LSM operations and even to guide improved urban planning. In STP, the water bodies associated with construction had high correlation with pupae positivity. The drivers of mosquito emergence in STP (defined as the multiplication of the abundance of these water bodies and their positivity rate) were puddles and channels (Table S3).

**Table S3: Distribution of water bodies according to type.**

| Baseline (prior to treatment) | | | | | | Treated water bodies | | | |
| --- | --- | --- | --- | --- | --- | --- | --- | --- | --- |
| Water body type | Number of water bodies | Number of samples | Pupa positive samples | Positivity rate | CI Wilson | Number of samples | Pupa positive samples | Positivity rate | CI Wilson |
| Puddle | 4,412 (35%) | 215 | 14 | 6.51% | 5.11% 8.26% | 4695 | 67 | 1.43% | 1.27 %1.60% |
| Channel | 2,920 (23%) | 324 | 26 | 8.02% | 6.73% 9.54% | 3834 | 41 | 1.07% | 0.93 %1.24% |
| Swamp | 2,041 (16%) | 121 | 6 | 4.96% | 3.42% 7.14% | 2687 | 32 | 1.19% | 1.01 %1.40% |
| Construction | 906 (7%) | 75 | 21 | 28.00% | 23.45% 33.05% | 1250 | 22 | 1.76% | 1.45% 2.14% |
| Pond | 626 (5%) | 49 | 4 | 8.16% | 5.21% 12.57% | 934 | 2 | 0.21% | 0.11% 0.41% |
| Tracks | 574 (4%) | 24 | 2 | 8.33% | 4.43% 15.14% | 677 | 7 | 1.03% | 0.73% 1.46% |
| Agriculture | 411 (3%) | 11 | 3 | 27.27% | 16.79% 41.07% | 299 | 6 | 2.01% | 1.38% 2.91% |
| Fringe | 389 (3%) | 38 | 4 | 10.53% | 6.74% 16.07% | 484 | 3 | 0.62% | 0.36% 1.05% |
| Others | 509 (4%) | 8 | 0 | 0.00% | 0.00% 9.76% | 588 | 8 | 1.36% | 0.98% 1.88% |
| **Total** | **12,788**  **(100%)** | **865** | **80** | **9.25%** | **8.37% 10.21%** | **15,448** | **188** | **1.22%** | **1.14% 1.30%** |

Tables S4 and S5 present the correlation of certain water body characteristics to pupa positivity before and after treatment.

Interestingly, although water pollution was negatively correlated to pupa positivity before treatment, it is positively correlated to pupa positivity after treatment. This may indicate that fieldworkers were not sufficiently trained to treat polluted water bodies with an added amount of *Bti*, as recommended by the manufacturer. Another interesting result relates to the durability of *bti* in different water bodies. Water bodies associated with construction maintained a relatively high positivity rate even after treatment.

**Table S4: Logistic regression of characteristics of water bodies and positivity before treatment.**

| Dep. variable | Pupa positivity | Observations: 865 |  |
| --- | --- | --- | --- |
|  |  |  |  |
|  |  |  |  |
|  |  |  |  |
|  | coef | CI | P |
| const | -1**.**7625 | -2.25 -1.27 | 0 |
| isPolluted | -0**.**3908 | -0.87 0.09 | 0. 108 |
| isShaded | -0**.**5619 | -1.06 -0.06 | 0**.**027 |
| isVegetation | -0**.**3226 | -0.85 0.2 | 0**.**227 |
| isTemporary | -0**.**7578 | -1.31 -0.2 | 0**.**007 |
| depth (cm) | 0**.**0150 | 0.01 0.02 | 0**.**002 |
| area (meter) | -0**.**0398 | -0.1 0.02 | 0**.**18 |

**Table S5: Logistic regression of water bodies characteristics and positivity after treatment**

| Dep. variable | Pupa positivity | No. Observations: 23,607 |  |
| --- | --- | --- | --- |
|  |  |  |  |
|  |  |  |  |
|  | **coef** | **CI** | **p** |
| **const** | -4**.**309 | -4.56 -4.06 | 0 |
| **isPolluted** | 0**.**2635 | 0.04 0.49 | 0**.**021 |
| **isShaded** | -0**.**1307 | -0.35 0.09 | 0**.**239 |
| **isVegetation** | -0**.**1204 | -0.35 0.11 | 0**.**297 |
| **isTemporary** | -0**.**0053 | -0.23 0.22 | 0**.**963 |
| **depth (cm)** | 0**.**0026 | -0.001 0.006 | 0**.**273 |
| **area (meter)** | -0**.**0042 | -0.03 0.02 | 0**.**763 |

Fig. S1 shows that the positivity of construction sites is highly correlated with the time elapsed from last treatment. Similar analysis shows that water bodies exposed to the sun (not shaded) also experience a relatively high correlation between the time elapsed from last spraying and the positivity rate, potentially due to degradation of the *bti*.


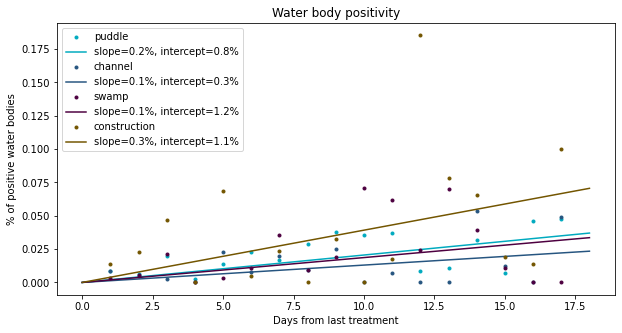


**Fig**. **S1: Water body positivity based on type**.
